# Lectures on the Ordinary Agents of Life, as Applicable to Therapeutics and Hygiene

**Published:** 1835-01-01

**Authors:** 


*Lectures on the Ordinary Agents of Life, as applicable to Therapeutics and Hygiene.* By Alexander Kilgour, M.D.—Edinburgh and London, 1834. 8vo. pp. 359.

We recollect, some years since, reading the travels of a hard-working, pains-taking German, one Dr. Stein. Among other places, Dr. Stein visited London, and, among other observations upon that celebrated city, he remarks, that there are no bells that can be pulled outside the houses. Whether this unaccountable assertion was the offspring of a bad memory, or whether the doctor has a special amaurosis with regard to those metallic processes called bell-handles, we know not; but certain it is that the observation stands in his book, and may be found by any one who will take the trouble to read his "Reisen nach den vorzüglichsten Hauptstädten von Mittel-Europa." Now, Dr. Kilgour is afflicted with a like cecity in the matter of hygienic precepts: the profession know little or nothing, he says, about the advantages of fresh air and regulated diet; and, if you want to pose your doctor most cruelly, you should ask him a question about roast or boiled—*teste Kilgouro.*

"A free ventilation might soon put him on his legs, and it would for certain expel effluvia; but open windows let in the cool air, and cold air is better felt than contagious effluvia; so the windows being kept shut, and the bed-curtains drawn close, the patient has the happiness of dying in an atmosphere of his own creating, raised to a proper putrifying temperature by means of a blazing sea-coal fire. What can we think of the man who, in circumstances like these, calls for paper, pen, and ink, in order that he may scrawl a receipt in indifferent Latin for worse medicine, and knows not to order that which would relieve the patient without pain or expense? He is not only guilty of the patient's death, but of his death aggravated by severe torments. Fortunately, there are occasions when this ignorance is discovered; and he who piques himself on knowing thoroughly the patient's complaint, and on writing a most scientific prescription for it, often looks blank enough when the simple but important question is put, What is the patient to be allowed to eat?

"Having therefore seen where we will most likely discover the cause, and not only this, but furnish the cure, or, at any rate, a most powerful ally or enemy of the disease, it must appear singular that so little attention should be given to this branch of science." (P. 4.)

Again, it is a capital thing to be curried: we do not mean in a dish with the Pulv. Pip. Cayenn. comp., but to have your skin curried with a brush. Every one knows this: Celsus knew it; Bichat knew it; the people who shampoo and are shampooed at Brighton are quite aware of it; in short, to use a familiar phrase, all the world is up to it, except one deaf and blind set of men—your learned physicians. They turn up their noses at anything that does not come out of the chemist's shop, and believe that Hygeia, like Truth, is only to be found at the bottom of that small well, a draught phial. Here is our authority.

"What makes the jockey so careful to curry his horse, but that he knows it is necessary for his beauty, his fleetness, his strength, and his health; for the horse exudes a large quantity of solid matter in his perspiration, and this retained in his hair would soon destroy him were the currying comb not frequently used. The professed 'Rubber' often meets with the *nasus aduncus* of the school-learned physician; but it would be well if this last learned gentleman would turn his scholarship to reading the many histories of cures by means of friction; and that he would recollect what Fuller says, that 'Exercise is to physic as a bandage is to surgery, an assistance or medium, without which many other administrations, though ever so noble, will not succeed.' Your regular pill, powder, and draught gentleman has a great contempt for rubbing; the effect of his ignorance." (P. 200.)

Now we believe that medical practitioners, so far from being ignorant of these matters, are remarkably well versed in them, and that Dr. Kilgour, in addressing his work to them, has acted as Dr. Stein would have done, had he imported a few tons of bell-metal into London, forgetful of Birmingham.

The plain truth is, (in spite of the author's protestations,) that these lectures are intended for unprofessional persons; and we think that this may form an amusing book enough for them to turn over at breakfast-time, when parliament is not sitting, and the newspaper is more than usually dry. The last lecture is needlessly and flippantly coarse, and will cause many to think but lightly of Dr. Kilgour's understanding. What philosopher, or what physician, ever before discussed the intercourse of the sexes in such a tone?

As we do not wish to part from our author in ill humour, we will conclude by extracting his account of training, as being a good compendium of that athletic education.

"As an example of the great effect of exercise on the body, we may here notice the practice of training. The horse is trained for the race-course, as is also the jockey that is to mount him. The pace and the wind of the horse are astonishingly improved, and the rider can be brought to any weight. The champions of the ring are regularly trained previous to the fight, so that they may be in good or prime condition, that the bone may be strong, the muscle firm and hard, and the wind long. The most flabby and shaking sot of a taproom will be, in three or four weeks' training, made as pretty and powerful a man as can be seen of his inches. All this is achieved by pure air, by nourishing food, and by exercise. The trainer takes his man to an open, and if possible, a hilly country, he cleans out his stomach and his bowels once or twice with an emetic and a warm and resinous purgative, he takes him from bed every morning at six, and exercises him in walking, running, leaping, riding, or a part of all, for three or four hours at least, he then breakfasts him on a beef steak, stale bread or biscuit, and a little tea or milk. Exercise is again had recourse to, either as before, or with the gloves, the ball, the dumbells or quoits. The dinner is beef steaks, or joint of mutton, or lean chop, stale bread, and a little beer. Exercise is again followed for three or four hours in the open air, and then supper of steaks and stale bread. The bed is hard, and the length of sleep not above seven hours. He has no idleness of mind or body; he must be always occupied, and it is best to engage his mind with the exercises of his body. He eats but three meals a day, and the solids must be nothing but the lean of fat beef, mutton, or venison. The legs of fowls are sometimes allowed for variety, but no veal or pork. The meat must always be broiled and under-done. Stale bread is almost the only vegetable substance allowed; sometimes a little potato, but no herbs. Eggs are occasionally taken, but no cheese, nor butter, or fat of any kind. The quantity of solids allowed during the day will depend upon the stomach and constitution of the individual, but must always be rather below his usual quantity; seldom, in any case, above twenty-four ounces. No condiments are allowed, with the exception of salt, and that only in a very small quantity. Fluids are considered as injurious. No food is given in this form: and, for the purposes of alleviating thirst and supplying the necessary waste of the fluids of the body, soft spring water is the best. No spirits are allowed, but occasionally a little porter after dinner or supper. The quantity of fluid, of any kind, taken during the twenty-four hours, must not exceed, in all, three English pints. Exercise of that kind in which the trained person is to exhibit, must form a large part of his daily occupation." (P. 206.)

